# Changes in Out-of-Pocket Spending and Catastrophic Health Care Expenditures Associated With Medicare Eligibility

**DOI:** 10.1001/jamahealthforum.2021.2531

**Published:** 2021-09-10

**Authors:** John W. Scott, Pooja U. Neiman, Renuka Tipirneni, Zhaohui Fan, John Z. Ayanian

**Affiliations:** 1 Department of Surgery, University of Michigan Medical School, Ann Arbor; 2 National Clinician Scholars Program, University of Michigan, Ann Arbor; 3 Division of General Medicine, University of Michigan Medical School, Ann Arbor; 4 Institute for Healthcare Policy and Innovation, University of Michigan, Ann Arbor; 5 Editor, *JAMA Health Forum*

## Abstract

This study examines whether becoming eligible for Medicare is associated with less out-of-pocket health care spending and lower catastrophic health care expenditure risk.

## Introduction

Despite large gains in health insurance coverage after the Patient Protection and Affordable Care Act, adults younger than 65 years remain at substantial financial risk because of high out-of-pocket (OOP) costs for health care.^1^ However, the gains in financial risk protection associated with Medicare, which covers 60 million elderly and disabled adults, remain incompletely understood. In this study, we assessed the association between gaining Medicare eligibility and OOP health care spending and catastrophic health care expenditure (CHE) risk.

## Methods

This cross-sectional study followed the Strengthening the Reporting of Observational Studies in Epidemiology (STROBE) reporting guideline. The institutional review board of the University of Michigan deemed the study exempt from review and waived the requirement for informed consent because we used deidentified publicly available data from the Medical Expenditure Panel Survey for 2014 through 2018. The study cohort included all individuals aged 57 to 73 years who reported any health care charges in the previous 12 months. Following previous work,^1,2^ we used a second-order local polynomial regression discontinuity design to evaluate changes associated with Medicare eligibility at age 65 years. Outcomes included insurance coverage (any Medicaid, Medicare, any private or uninsured), mean annual income, mean annual medical charges, mean annual OOP health care spending (exclusive of premiums), CHE (defined as OOP spending ≥40% of annual income minus spending on food and housing),^3,4^ and delaying care because of affordability.

Because measured and unmeasured confounders should be balanced on either side of the age threshold, regression discontinuity estimates the isolated association between Medicare age eligibility and financial outcomes. The regression discontinuity analyses were limited to the 8 years preceding (ages 57-64) and the 8 years following (ages 66-73) the year that a patient becomes eligible for Medicare. We also ran sensitivity analyses using a first-order polynomial design, alternative age bandwidth choices, and excluding ages 64 to 66 years. Spending data were standardized to 2018 US dollars. Analyses were conducted from March 15 to June 28, 2021, using Stata, version 15.1 (StataCorp, LLC) and Medical Expenditure Panel Survey weights that accounted for the complex survey design. A 2-sided *P* < .05 was considered to be significant.

## Results

Among the 24 700 survey respondents aged 57 to 73 years, 91.7% incurred medical charges during the years surveyed (weighted 274 million person-years). The Figure shows the mean OOP health care spending and CHE rate by age. The Table provides mean results for key outcomes among respondents aged 64 years and 66 years. Notably, 8.6% of adults aged 64 years experienced CHE. Regression discontinuity analyses showed a −4.9 (95% CI, −5.3 to −4.6) percentage-point reduction in the uninsured rate. Despite a 5% increase in medical charges ($1194; 95% CI, $271-$2117) after age 65 years, OOP spending decreased by 27% (−$383; 95% CI, −$512 to −$254), and the CHE rate decreased by 35% (−3.0 [95% CI, −3.8 to −2.2] percentage points) (Table). The Table shows changes in OOP spending by type. Affordability-related delays in care decreased by 1.1 (95% CI, −1.6 to −0.8) percentage points. All 3 sensitivity analyses yielded similar results.

**Figure.**
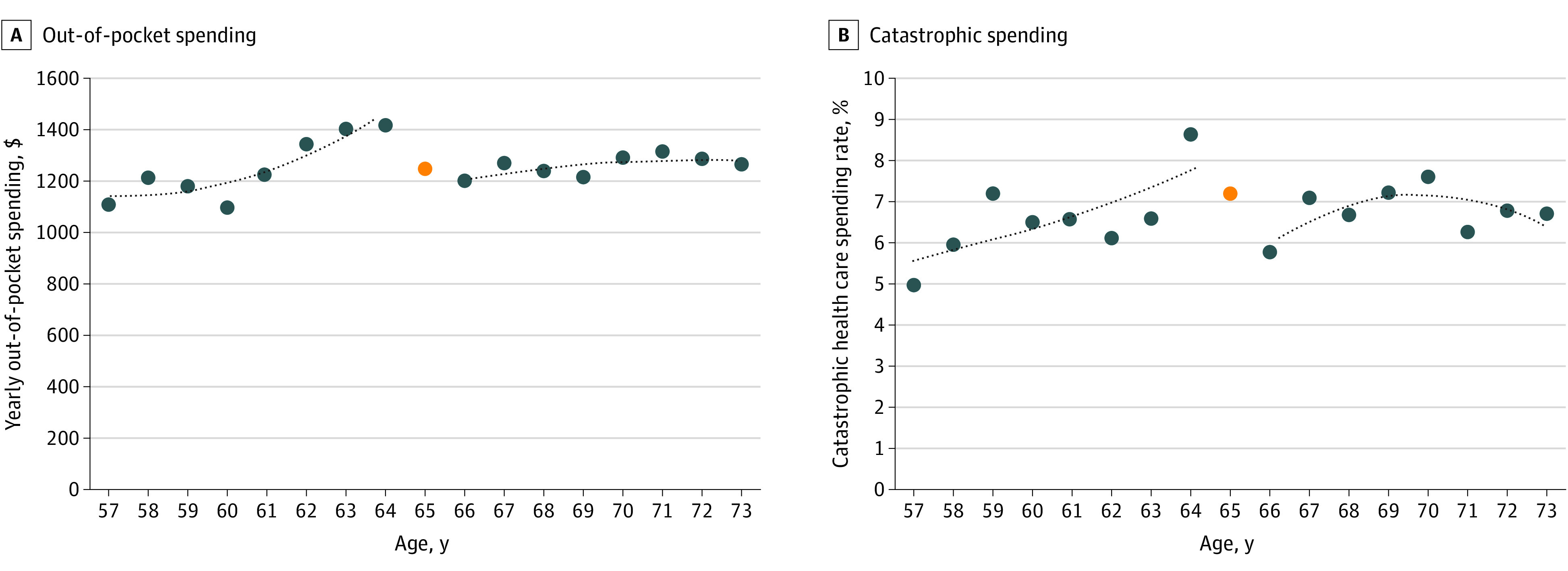
Health Care Spending Rates by Age From 2014 Through 2018 A and B, Data are from the Medical Expenditure Panel Survey. Lines represent second-order polynomial best-fit curves of data for respondents aged 57 to 64 years and aged 66 to 73 years (blue circles). Data for age 65 years (orange circles) are excluded because that information consists of respondents who were aged 64 years for 1 to 11 months of the recall period. Amounts illustrated in panel A have been converted to 2018 US dollars.

**Table.** Insurance Coverage and Affordability Measures Before and After Medicare Eligibility at Age 65 Years^a^

Variables	Unadjusted *t* test			Regression discontinuity at age 65 y^b^		
	Age 64 y	Age 66 y	*P* value	Absolute change (95% CI), percentage points	Relative change, %	*P* value
Uninsured, weighted %	5.0	0.2	<.001	−4.9 (−5.3 to −4.6)	−98	<.001
Any private insurance, weighted %	77.4	60.1	<.001	−20.2 (−22.5 to −17.9)	−27	<.001
Any Medicare, weighted %	13.5	96.9	<.001	81.7 (80.9 to 82.5)	605	<.001
Any Medicaid, weighted %	13.1	.8	<.001	−2.0 (−5.0 to 1.0)	−15	.19
Mean annual income, $	72 040	71 424	.74	506 (−1824 to 2837)	0.7	.67
Mean annual medical charges, $	24 801	26 443	.63	1194 (271 to 2117)	5	.01
Mean annual OOP health care spending, $^c^	1421	1204	.03	−383 (−512 to −254)	−27	<.001
Office-based visits	483	318	.02	−181 (−262 to −99)	−37	<.001
Outpatient spending	99	44	<.001	−56 (−79 to −34)	−56	<.001
Emergency department spending	45	17	.01	−19 (−28 to −10)	−42	<.001
Inpatient spending	47	37	.50	−31 (−60 to −2)	−66	.03
Other equipment	78	37	.09	−53 (−113 to 6)	−68	.08
Home health spending	3	8	.21	−23 (−57 to 11)	−838	.18
Prescription medications	345	371	.44	−19 (−98 to 59)	−6	.63
Dental and vision spending	321	372	.29	0 (−64 to 63)	0	.99
Catastrophic health care spending, weighted %	8.6	5.8	<.001	−3.0 (−3.8 to −2.2)	−35	<.001
Delay in getting necessary medical care because of cost/affordability, weighted %	6.3	6.1	.004	−1.1 (−1.6 to −0.8)	−17	<.001

Abbreviation: OOP, out of pocket.

^a^ Data are from the Medical Expenditure Panel Survey, 2014-2018. A two-sided *t* test was used to compare outcomes among patients aged 64 and 66 years.

^b^ Regression discontinuity analyses compare data for ages 57 to 64 years to that of ages 66 to 73 years.

^c^ Total out-of-pocket and catastrophic health care spending excludes spending on premiums.

## Discussion

We found that becoming eligible for Medicare at age 65 years was associated with a 27% reduction in OOP spending and a 35% reduction in the risk of catastrophic health care expenditures for older adults from 2014 through 2018. These findings extend those of previous work^4,5,6^ by quantifying the type of spending changes and magnitude of financial risk protection currently associated with Medicare. Despite the substantial health insurance coverage gains attributable to the Affordable Care Act, nearly 9% of adults aged 64 years still experienced CHE from 2014 through 2018. Interpretation of our results is subject to the limitations common to all observational data, namely the inability to demonstrate causal influence.

The substantial declines in OOP spending and the catastrophic spending rate that occur at age 65 years show the important role that Medicare plays in improving financial risk protection for older adults. The importance of improved financial risk protection is further supported by the substantial decline in delaying care because of cost or affordability. Taken together, these findings suggest that lowering the Medicare eligibility age may provide substantial benefit by mitigating financial strain and delays in care associated with unaffordable and burdensome health care expenses.
